# Prophage recombinases-mediated genome engineering in *Lactobacillus plantarum*

**DOI:** 10.1186/s12934-015-0344-z

**Published:** 2015-10-05

**Authors:** Peng Yang, Jing Wang, Qingsheng Qi

**Affiliations:** State Key Laboratory of Microbial Technology, Shandong University, Jinan, 250100 People’s Republic of China

**Keywords:** Homologous recombination, Lactic acid bacteria, Prophage, Genome modification, Marker-free

## Abstract

**Background:**

*Lactobacillus plantarum* is a food-grade microorganism with industrial and medical relevance belonging to the group of lactic acid bacteria (LAB). Traditional strategies for obtaining gene deletion variants in this organism are mainly vector-based double-crossover methods, which are inefficient and laborious. A feasible possibility to solve this problem is the recombineering, which greatly expands the possibilities for engineering DNA molecules in vivo in various organisms.

**Results:**

In this work, a double-stranded DNA (dsDNA) recombineering system was established in *L. plantarum*. An exonuclease encoded by *lp_0642* and a potential host-nuclease inhibitor encoded by *lp_0640* involved in dsDNA recombination were identified from a prophage P1 locus in *L. plantarum* WCFS1. These two proteins, combined with the previously characterized single strand annealing protein encoded by *lp_0641*, can perform homologous recombination between a heterologous dsDNA substrate and host genomic DNA. Based on this, we developed a method for marker-free genetic manipulation of the chromosome in *L. plantarum*.

**Conclusions:**

This Lp_0640-41-42-mediated recombination allowed easy screening of mutants and could serve as an alternative to other genetic manipulation methods. We expect that this method can help for understanding the probiotic functionality and physiology of LAB.

**Electronic supplementary material:**

The online version of this article (doi:10.1186/s12934-015-0344-z) contains supplementary material, which is available to authorized users.

## Background

*Lactobacillus plantarum* is a food-grade microorganism belonging to the group of lactic acid bacteria (LAB), many species from which are with industrial and medical relevance. It is involved in many food raw-material fermentations and is a commensal of the human gastrointestinal tract. Certain strains from this group are already used as probiotics or as delivery vehicles for therapeutic compounds [1, 2]. With the development of metabolic engineering and synthetic biology, many LAB species are now used as cell factories for the bioproduction of value-added chemicals [3, 4]. *Lactobacillus* species have an unusually high level of phylogenetic diversity, of which *L. plantarum* has one of the largest genomes, suggesting its ecological flexibility [5]. Precise and fluent genetic manipulation is vital for functional genomics in such species.

Traditional strategies for obtaining gene deletion variants in LAB are mainly vector-based double-crossover methods [6–8]. These methods involve integration of a vector containing a selectable marker and homologous sequences flanking the gene of interest into the genome, followed by resolution of the co-integrate. Both steps are mediated by endogenous RecA function. Integration vectors include non-replicating ones and conditionally replicating ones. However, due to low efficiency of vector excision and an inherent allelic replacement frequency of 50 % for the mutant genotype during the second step [6], such methods are often laborious when it comes to screening prospective mutants. Possible solutions may help including incorporating a counter-selectable genetic marker, or placing a marker between the homologous sequences followed by its elimination [9–11], but these methods are still performed in two steps (vector integration and co-integrate resolution) and it’s still no easy to screen prospective mutants. Transposon mediated insertional inactivation is efficient, but the insertion loci are random.

Red/RecET-mediated dsDNA recombination has greatly facilitated rapid and precise functional genomic analysis in *Escherichia coli* as well as several other organisms [12–16]. The Red recombination proteins are encoded by three adjacent genes, *gam*, *bet* and *exo*, in λ phage, while RecET are encoded by two adjacent genes, *recE* and *recT*, in Rac prophage. The λ Exo/Beta and RecE/RecT protein pairs are functional analogs although not related at the sequence level. Exo and RecE are 5′–3′ dsDNA-dependent exonucleases that generate 3′-ended single-stranded DNA (ssDNA) overhangs [17, 18]; Beta and RecT are single strand annealing proteins (SSAP) that promote annealing of complementary DNA strands, strand exchange and strand invasion [19, 20]. The SSAPs themselves are sufficient to facilitate ssDNA recombineering [21]. Gam prevents degradation of linear dsDNA introduced into the cell by inhibiting two potent host nucleases, RecBCD and SbcCD, thus enhancing the recombination efficiency of the Red/RecET system [22–24]. However, the Rac prophage does not encode a functional analogue of Gam.

Applying the Red/RecET system in bacteria other than *E. coli* resulted in only limited success, which may be due to a requirement for specific interactions between the recombinases and host-encoded proteins [25, 26]. With this in mind, it is expected that recombinase proteins mediate recombination more efficiently in their native or closely related hosts [12]. Recently, ssDNA recombineering system was developed in *Lactobacillus reuteri* and several other LAB strains [27–29]. However, dsDNA recombineering system for LAB strains is absent. While ssDNA recombineering system is efficient for subtle modification of the genome (e.g., point mutations, RBS/promoter substitution or premature translation termination), dsDNA recombineering system holds certain advantages in large genomic region manipulation including gene insertion.

In this study, we identified potential analogs of Gam, Beta and Exo in *L. plantarum* WCFS1 based on similarity and location. Lp_0640, Lp_0641 and Lp_0642 were demonstrated to efficiently perform homologous recombination between a heterologous dsDNA substrate and host genomic DNA. Combined with the *loxP*/Cre system, a marker-free genetic manipulation method in *L. plantarum* was developed. This method allowed easy screening of mutants and may help us to understand the probiotic functionality and physiology of LAB.

## Results

### Identification and characterization of Lp_0640 and Lp_0642

The Red system from λ phage has been widely used for genetic manipulation in *E. coli* and several other organisms including *Salmonella enterica* and *Agrobacterium tumefaciens* [25, 26]. However, this system has not been effectively applied in *L. plantarum* probably due to host-specific interactions (data not shown).

**Fig. 1:**
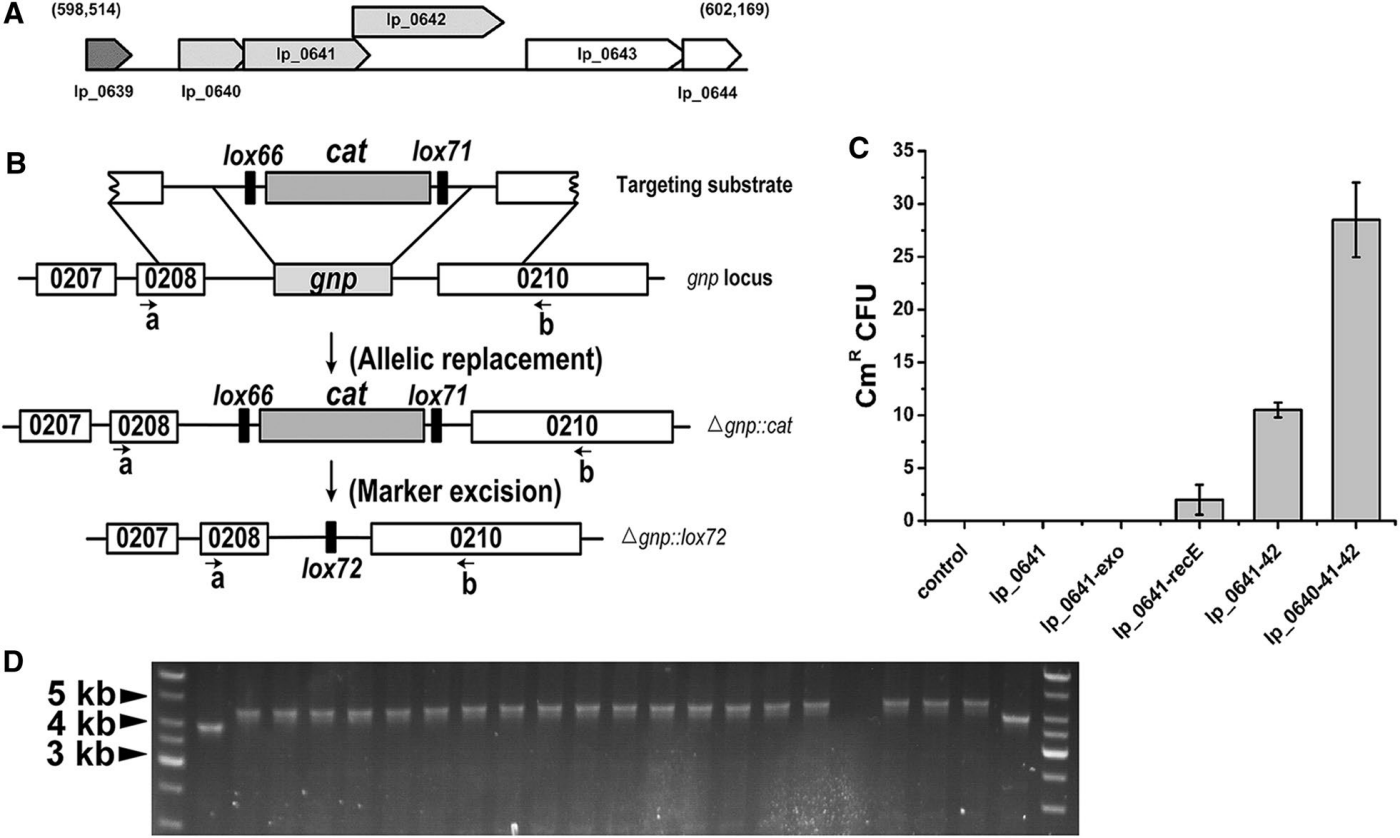
Characterization of the *lp_0640*-*41*-*42* operon. **A** Layout and genetic context of the *lp_0640*-*41*-*42* operon. The *numbers* in *parentheses* indicate the genome locus. **B** Schematic diagram showing in-frame *gnp* deletion and marker elimination. Lp_0640-41-42 mediated allelic replacement resulted in disruption of the *gnp* gene and insertion of the *cat* marker, while Cre subsequently eliminated the marker. **C** dsDNA recombination assay with different protein combinations. 1 μg dsDNA substrate was electroporated into *L. plantarum* JDM1 expressing various combinations of proteins, and the respective resultant chloramphenicol resistant colony number were shown. pSIP411 was used as the control. Results are the averages from at least three independent experiments, with standard deviations indicated by *error bars*. **D** Inspection of potential *Δgnp::cat* mutants by PCR testing using primers gnp-testA (*a*) and gnp-testB (*b*), as shown in **B**. Lanes *1* and *24* show DNA ladders, lanes *2* and *23* the wild-type strains expected to generate amplicons of ∼3.9 kb. Lanes *3*–*22* were tested colonies, with correct mutants expected to generate amplicons of ∼4.3 kb

In a previous study, a RecT/Beta analogue was identified in *L. plantarum* WCFS1, which has 46 % identity with the known *L. reuteri* RecT [29]. The coding gene of this protein, *lp_0641*, is an ORF in prophage P1 (a 44-kb temperate *pac*-site phage) [30]. Most recombinase proteins are encoded by bacterial phages, prophages or their remnants, as is the case for Red and RecET in *E. coli*, and SSAP–exonuclease pairs always lie next to each other. After further inspection of prophage P1, we found *lp_0641*, *lp_0640* and *lp_0642* could transcript as an operon (Fig. 1A), as indicated in the MetaCyc Metabolic Pathway Database (http://metacyc.org/). *lp_0642* overlapped by 77 nucleotides (nt) with *lp_0641* and encodes a 286-residue protein. BLAST search of this protein showed specific hits to the DUF3799 conserved domain (proteins of this family are likely to be nucleases) over an 80 % query coverage [31]. *lp_0640* encodes a 128-residue protein similar to Gam (138 residues) in size.

The convincing SSAP identity of Lp_0641 and the same 5′–3′ gene arrangement between the Lp_0640-41-42 and Red operons suggested that Lp_0642 is a candidate exonuclease functionally similar to Exo, while Lp_0640 is a Gam analogue. However, Lp_0640-41-42 does not show any identity with λ Red/Rac proteins at either the nucleotide sequence or amino acid sequence levels, which suggests different origins of the recombinases.

To avoid the interference of other proteins in the host, functions of Lp_0640-41-42 were evaluated using a dsDNA recombination assay heterologously in *L. plantarum* JDM1. A mutagenesis vector for *gnp* inactivation (Additional file 1: Figure S1) was constructed containing a *lox66*-*cat*-*lox71* cassette conferring chloramphenicol resistance, flanked by 1.4-kb fragments from either side of the genomic *gnp* (a non-essential gene coding for glucosamine-6-phosphate isomerase) target locus. *lox66* and *lox71* sites were introduced for subsequent Cre-mediated removal of the *cat* marker [32]. A dsDNA substrate containing the flanks and *cat* marker was then generated by PCR from the vector, digested with *Dpn*I to eliminate methylated template DNA and electroporated into *L. plantarum* JDM1 expressing different protein combinations. An inducible expression vector pSIP411 was chosen to prevent potential toxic effects exerted by recombinase proteins. Allelic replacement would result in disruption of the *gnp* gene and insertion of the *cat* marker, and recombinants with allele replacement would grow on chloramphenicol-containing selective medium (Fig. 1B). On analysis of the resulting strains, *lp_0641*-*recE* and *lp_0641*-*42* expression mediated dsDNA recombination, while *lp_0641* and *lp_0641*-*exo* expression did not (Fig. 1C). A functional pair of SSAP and dsDNA-dependent exonuclease is necessary for dsDNA recombination, so this result suggests again that Lp_0642 is a RecE analog. Introduction of *lp_0640* greatly enhanced the recombination efficiency, which suggested that Lp_0640 is a potential Gam analogue. The efficiency of *lp_0641*-*42*-mediated dsDNA recombination was higher than that of *lp_0641*-*recE*, and *lp_0641*-*exo* did not function, which may suggest host-specific interactions. The fidelity of the recombinants was checked by PCR using primers gnp-testA and gnp-testB. 20 colonies were randomly selected and subjected to test; 19 of them showed the mutant genotype, indicating the occurrence of simple allele replacement at the target locus (Fig. 1D).

These results further indicated that *lp_0640*-*41*-*42* encodes a presumptive host exonuclease inhibitor, a SSAP and a dsDNA exonuclease from 5′ to 3′. The combination *lp_0640*-*41*-*42* was chosen for further study due to its relatively high recombination efficiency.

The non-replicating *gnp* mutagenesis vector constructed was also used directly to perform gene deletion via the double-crossover method. We got 16 Cm^R^ colonies in total in three independent experiments (7, 4, 5 colonies respectively), and none of these colonies corresponded to the *Δgnp::cat* genotype. This means that single crossover must have occurred through either homology A or homology B, and resultant strains should be cultivated for about 200 generations (more than 10 days) under non-selection condition to allow a second recombination event. After the second recombination event, strain could have either the wild genotype or the prospective mutant genotype (1:1 theoretically), which was determined by the homology arm used. After single colony isolation, 200 colonies were tested for loss of the *cat* marker and 7 colonies turned to be Cm^S^. Of the 7 Cm^S^ colonies, 5 were shown to be the wild genotype and 2 were correct mutant genotype.

Considerations should be taken that if target gene deletion would bring growth shortage, the efficiency would be lower. On this point, Lp_0640-41-42-mediated recombination allowed easy screening of mutants and simplified the selection process.

### Optimization of the in vivo recombination process

**Fig. 2:**
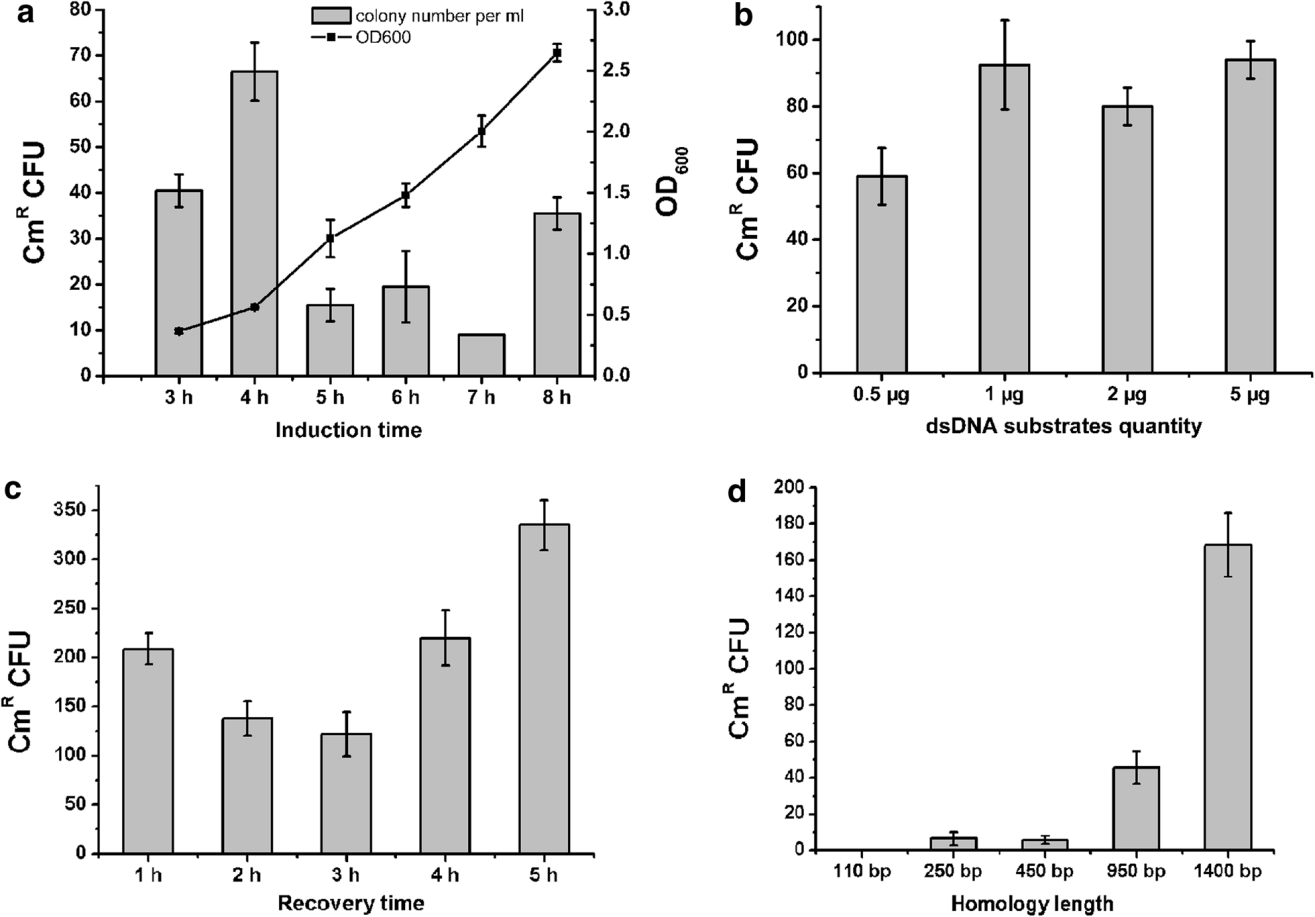
Optimization of dsDNA recombination parameters. **a** The effect of the induction time on selection efficiency. The culture was incubated for 3–8 h before addition of the inducing peptides. 0.5 μg dsDNA substrates with 1.4-kb homologies were used for electroporation and the recovery time was 3 h. OD_600_ of the culture on induction is also shown. **b** The effect of the quantity of dsDNA substrates on selection efficiency. The culture was incubated for 4 h before addition of the inducing peptides. 0.5–5 μg dsDNA substrates with 1.4-kb homologies were used for electroporation and the recovery time was 3 h. **c** The effect of the recovery time on selection efficiency. The culture was incubated for 4 h before addition of the inducing peptides. 1 μg dsDNA substrates were used for electroporation and the recovery time was 1–5 h. **d** The effect of the homology length of dsDNA substrates on selection efficiency. PCR products with various homology lengths were generated by PCR and added at constant molarity. The culture was incubated for 4 h before addition of the inducing peptides. 1 μg dsDNA substrates was used for electroporation and the recovery time was 1 h. Results are the averages from at least three independent experiments, with standard deviations indicated by *error bars*

To enhance the mutants selection efficiency, we examined induction time of the recombinases from 3 to 8 h. 0.5 μg dsDNA substrates with 1.4-kb homologies were used for electroporation and the recovery time was 3 h. As a result, induction at 4 h, when the OD_600_ was 0.56, yielded the most recombinants (Fig. 2a). This OD_600_ is consistent with the optima for electrocompetent-cell preparation. Then, we determined the effect of substrate concentration on the selection efficiency by varying the amount of dsDNA (0.5–5 μg) added to the electroporation mix. Higher frequency was observed when the substrate concentration was increased from 0.5 to 1 μg, but further increase did not improve the efficiency (Fig. 2b), so 1 μg substrate concentration was used further. Essentially, allele replacement occurs during recovery cultivation. Therefore, extending the recovery cultivation time may increase the selection efficiency. However, fewer recombinants appeared as the incubation time was extended from 1 to 2 or 3 h (Fig. 2c). For 5 h, more Cm^R^ colonies appeared due to an increase in viable cells (the ratio between Cm^R^ colonies and viable cells did not increase, at about 35 ± 4 Cm^R^ colonies/10^8^ viable cells), but to save time, 1 h was applicable. The 1 h optimum recovery was short compared with that following plasmid-electroporation, which is generally 2–3 h.

Because 50-bp homology length is enough for Red/RecET recombineering in *E. coli*, we assessed the possibility of using short homology for Lp_0640-41-42-mediated recombination. Substrates with various homology lengths (110–1400 bp) were generated by PCR from plasmid pLPM-gnp and added at constant molarity. There was a positive correlation between the recombination frequency and the homology sequence length in the size range 110 bp–1.4 kb and the efficiency was rather low when the homologies were shorter than 1 kb (Fig. 2d).

### Targeting other genomic loci

Genomic loci other than *gnp* were also tested for Lp_0640-41-42-mediated recombination. First, gene *ldhD* coding for D-lactate dehydrogenase was selected. As described above, a mutagenesis vector was constructed with 1-kb flanks. This homology length was chosen for easy vector construction as well as an acceptable recombination efficiency (Fig. 2d). With 1 μg dsDNA substrate, we obtained about 40 colonies per plate. Twenty colonies were randomly selected and tested, of which all showed the correct genotype. Then an operon of 8 kb (the *glg* operon encoding functions related to glycogen metabolism) was chosen as a second target. As designed, homologous recombination at the target locus would lead to a 6-kb deletion within this operon. In a first trial, with 1 μg substrate (1-kb flanks), we only obtained three colonies. Although PCR assay showed that they were all correct recombinants, the efficiency was rather low. A possible reason was the degradation of the dsDNA substrate by a host nuclease. To protect substrate DNA, we modified one 5′ end of the dsDNA substrate corresponding to the lagging strand of the homology arm with phosphorothioate bonds [33]. Using this method, the colony number reached 30 per plate.

### Insertion of *gusA* into the genome

**Fig. 3:**
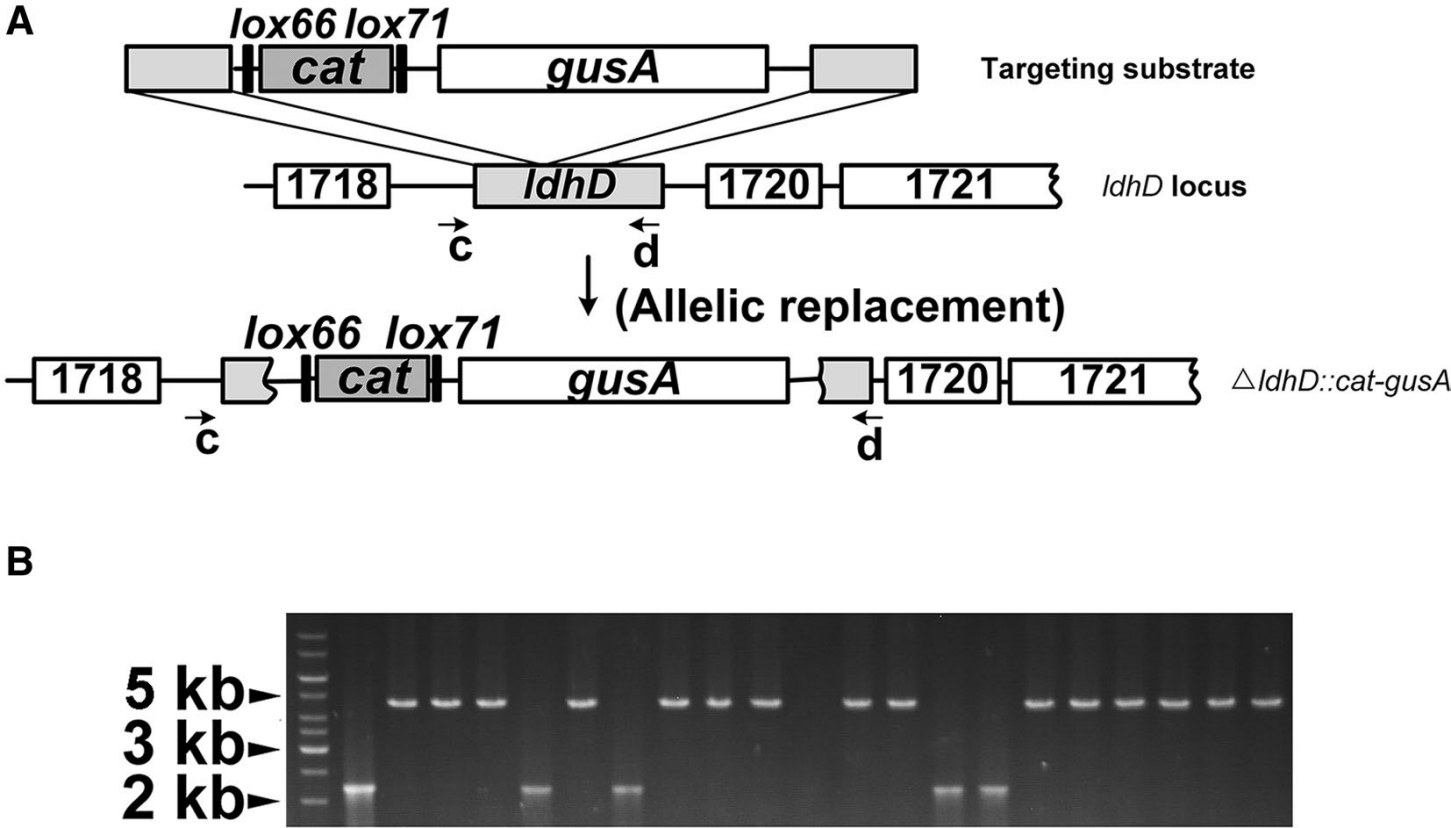
Insertion of *gusA* into the genomic *ldhD* locus. **A** Schematic diagram illustrating *gusA* insertion. The substrate contained ~1-kb flanks located next to each other on the genome. Allelic replacement resulted in disruption of the *ldhD* gene and simultaneous insertion of the *cat* marker and *gusA* gene. In theory, any nonessential locus can be targeted. Primers ldhD-testA (*c*) and gus-testB (*d*) used for PCR testing are shown. **B** Inspection of potential mutants by PCR testing using primers *c* and *d*, as shown in **A**. Lane *1* shows DNA ladder and lane *2* the wild-type strain expected to generate an amplicon of ∼2 kb. Lanes *3*–*22* were tested colonies, with correct mutants expected to generate amplicons of ∼4.7 kb

Lp_0640-41-42-mediated gene insertion was also tested. Different from the gene-deletion cases described above, the homology arms are adjacent on the genome for gene-insertion. The *gusA* gene from *E. coli*, coding for β-d-glucuronidase, was chosen because there is a lack of such enzymatic activity in *L. plantarum*. Recombinants with GusA activity should be readily screenable on plates with X-Gluc-containing medium (positive colonies resulting in a white to blue change) [34]. Similarly, a mutagenesis vector for *gusA* insertion (Additional file 1: Figure S1) was constructed followed by PCR and Lp_0640-41-42-mediated recombination. Allelic replacement would result in disruption of the *ldhD* gene and simultaneous insertion of the *cat* marker and *gusA* gene (Fig. 3A). As a result, we obtained 35 Cm^R^ colonies in total in three independent experiments using 1 μg phosphorothioated dsDNA substrate (1-kb flanks). 20 of them were selected and tested by PCR, of which 15 were correct (Fig. 3B).

Unexpectedly, *ΔldhD::gusA* colonies did not turn blue on X-Gluc-containing media. Three mutants were randomly selected and sequenced, and all showed the correct genotype. To confirm the Gus enzyme was properly expressed, we used Gus buffer to test the three mutants, and their precipitates suspended in Gus buffer turned blue while the wild strain did not (Additional file 2: Figure S2). That these mutants did not turn blue on media containing X-Gluc may be due to a weak RBS.

### Cre based selectable marker excision

**Fig. 4:**
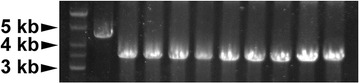
Cre-mediated excision of the selectable marker. After Cre induction and incubation, potential *Δgnp::lox72* mutants were inspected by PCR testing using primers gnp-testA and gnp-testB. Lane *1* shows DNA ladder, lane *2* was the *Δgnp::cat* strain. Lanes *3*–*11* were tested colonies, with marker-free mutants expected to generate amplicons of ∼3.2 kb

To eliminate the *cat* selection marker after recombination, the *loxP*/Cre system was employed. The phage protein Cre catalyzes site-specific recombination between two of its recognition sites, *loxP*. DNA sequence flanked by *loxP* sites would be excised when the *loxP* sites are convergently oriented and inverted when the *loxP* sites are divergently oriented [35]. To minimize genetic instability, convergently oriented *lox66*/*lox71* sites were used in our experiments. The recombinase-expressing plasmid pLP-gba was eliminated by culturing a *Δgnp::cat* mutant in the absence of erythromycin selection for 24 h. Then the Cre helper plasmid pLP-cre was introduced. After Cre induction and incubation, single colonies were checked by PCR using primers gnp-testA and gnp-testB. Recombination between *lox66* and *lox71* would lead to excision of the *cat* marker gene and form a *lox72* site that is poorly recognized by Cre (Fig. 1B). On analysis, all of the nine tested colonies showed the mutant genotype and could not grow on chloramphenicol-containing media (Fig. 4). Finally, the Cre helper plasmid was cured by culturing a marker-free mutant for 24 h in the absence of erythromycin selection.

**Fig. 5:**
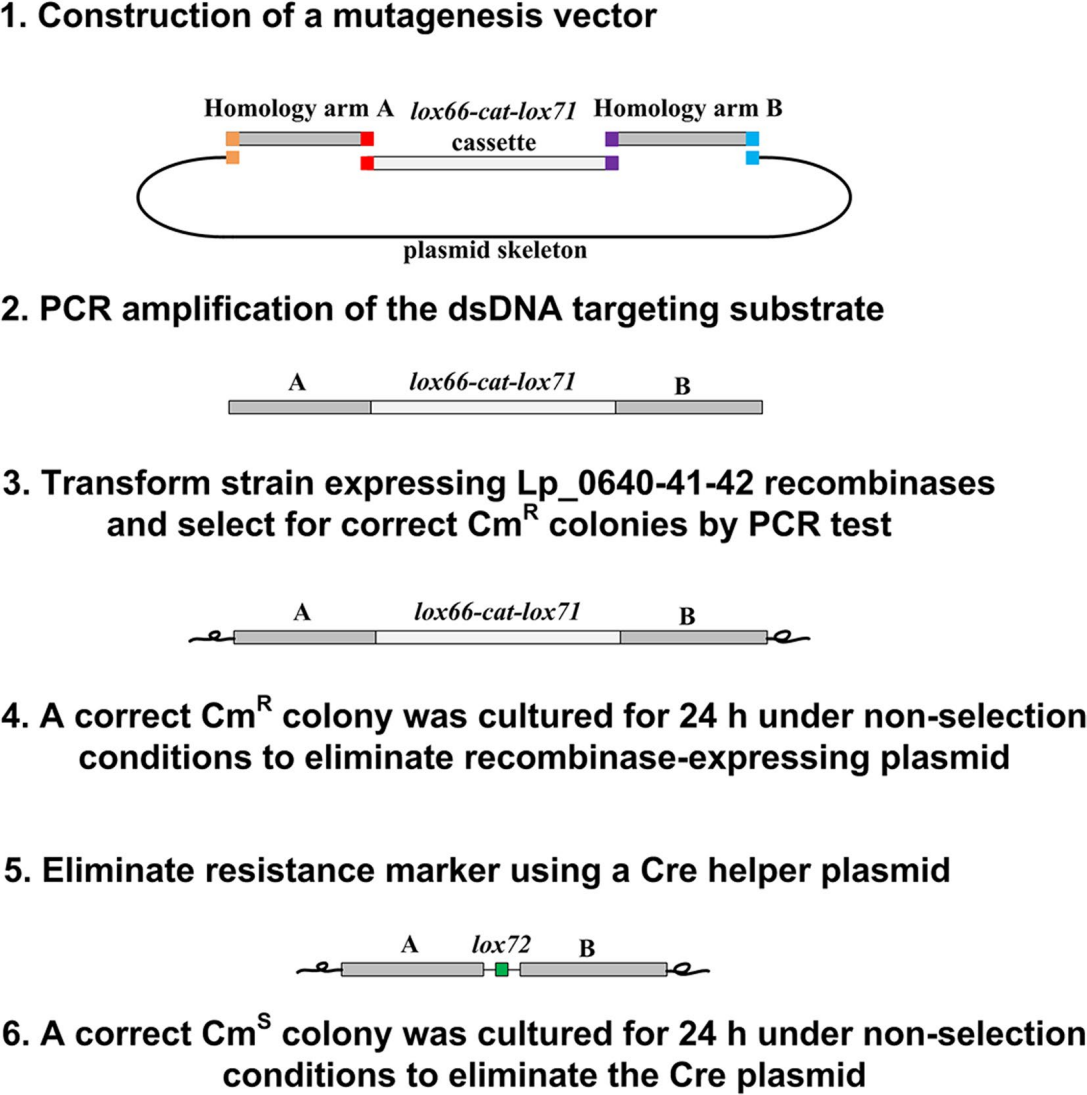
A general procedure for Lp_0640-41-42-mediated genome engineering. (*1*) A mutagenesis vector containing a *lox66*-*cat*-*lox71* cassette flanked by 1-kb homologies was constructed. Homology arm A and Homology arm B were generated by PCR of the upstream and downstream of the target locus in the genome. DNA fragments sharing terminal sequence overlaps (indicated as *colored-squares*) were assembled via Gibson method. (*2*) dsDNA substrate was generated from the mutagenesis vector by PCR and subjected to *Dpn*I digestion to eliminate the plasmid template. (*3*) Lp_0640-41-42-mediated recombination was performed to edit the genome of strains and correct mutants could be screened out by antibiotic selection and PCR test. (*4*) The recombinase-expressing plasmid was eliminated by culturing a correct mutant derived in procedure 3 in the absence of erythromycin selection for 24 h. (*5*) The Cre helper plasmid was used to excise the *cat* marker, and a *lox72* site (34 bp) that is poorly recognized by Cre was formed. (*6*) The Cre plasmid was eliminated by culturing a correct mutant derived in procedure 5 in the absence of erythromycin selection for 24 h. The resultant strain was free of any plasmid or genetic selection marker

Thus, a marker-free method combining recombineering and the *loxP*/Cre system for genetic manipulation in *L. plantarum* was developed (Fig. 5). By changing the *lox66*-*cat*-*lox71* cassette, this method may also find application in gene insertion, promoter swapping and other genetic manipulations.

With this method, efficiency of Lp_0640-41-42-mediated dsDNA recombination in strain *L. plantarum* WCFS1 was assessed. Using *nagB* (the functional equivalent gene of *gnp* in *L. plantarum* JDM1) as the target, the Cm^R^ colony number per ml recovery culture (2000 ± 132) was about 50 times higher in WCFS1 strain with pLP-gba than that in JDM1, while the wild WCFS1 strain without the recombinase-expressing plasmid did not generate any Cm^R^ colonies. We inspected the electroporation efficiency of both strains and found that WCFS1 was more accessible to foreign DNA (electroporation efficiency: 6.1 × 10^−3^ vs 3.4 × 10^−6^ for WCFS1 and JDM1 respectively). So the low selection efficiency in JDM1 was at least partially due to the lower electroporation efficiency. The native *lp_0640*-*41*-*42* operon within the P1 prophage may be silent or too weak to mediate recombination.

### Potential recombinases with similarity to Lp_0640-41-42 in other species

Because of specific interactions between the recombinases and host-encoded factors, individual recombinases appear to function only in species closely related to their origin. Using *L. plantarum* Lp_0640, Lp_0641 and Lp_0642 as initial query sequences, we searched the NCBI NR database with PSI-BLAST for strains that contain both a potential SSAP and an exonuclease partner [36]. Predicted proteins were mainly from the genera *Lactobacillus* and *Enterococcus*. Proteins from strains *L. paraplantarum* and *L. pentosus* IG1 shared the highest identity (~90 %) with Lp_0640-41-42, and these proteins have a common phage P1 origin. Only one Lp_0640 homolog was found in *L. paraplantarum* (96 % identity). For the strains that lacked a potential host exonuclease inhibitor, recombineering systems may also be developed since host exonuclease inhibitor was not essential for dsDNA recombination [24]. However, there are genes in the same operons as the predicted SSAP and exonuclease pairs in many strains, and these genes have similar size to *lp_0640* (Table 1). These may also be host exonuclease inhibitors which could enhance recombination. Further studies are needed to test the feasibility of Lp_0640-41-42 and the proteins listed in Table 1 for recombination in other related species.

**Table 1:** Proteins with similarity to *L. plantarum* Lp_0640, Lp_0641 and Lp_0642

Source organisms	Lp_0640	Lp_0641		Lp_0642	
	Gene	Gene	Identity (%)	Gene	Identity (%)
*L. paraplantarum*	*HR47_13610*	*HR47_13605*	96	*HR47_13600*	89
*L. pentosus* IG1	*LPENT_03271* ^a^	*LPENT_03270*	95	*LPENT_03269*	84
*L. paracasei* N1115		*AF91_11175*	56	*AF91_11170*	54
*L. rhamnosus* LRHMDP2	*LRHMDP2_1610* ^a^	*LRHMDP2_1611*	56	*LRHMDP2_1612*	56
*L. buchneri* NRRL B-30929	*Lbuc_1445* ^a^	*Lbuc_1444*	55	*Lbuc_1443*	48
*L. brevis* KB290	*LVISKB_1732* ^a^	*LVISKB_1731*	40	*LVISKB_1730*	53
*L. casei* 12A		*LCA12A_0940*	43	*LCA12A_0939*	56
*L. fermentum* 3872		*N573_02575*	44	*N573_02570*	42
*L. reuteri* JCM 1112		*LAR_0775*	45	*LAR_1074*	45
*S. pyogenes*		*FE90_0912*	43	*FE90_0180*	44
*S. agalactiae* CCUG 91		*SAG0049_02245*	45	*SAG0049_02250*	44
*E. faecalis* 599		*HMPREF1327_00926*	47	*HMPREF1327_00925*	45
*E. faecium* EnGen0024	*OK7_04346* ^a^	*OK7_04347*	40	*OK7_04348*	43
*E. mundtii* QU 25	*EMQU_0764* ^a^	*EMQU_0765*	42	*EMQU_0766*	45
*E. hirae* EnGen0127		*SE1_00426*	40	*SE1_00427*	45
*E. flavescens* ATCC 49996		*I582_02415*	41	*I582_02414*	44
*E. raffinosus* ATCC 49464		*I590_00671*	40	*I590_00670*	44

^a^ Genes that locate within the same operon as their potential SSAP and exonuclease partner but not related with Lp_0640 at either nucleotide sequence or amino acid sequence level. These genes are similar to *lp_0640* in size

## Discussion

In this study, we identified and characterized a prophage P1 operon from *L. plantarum* WCFS1 that encodes three proteins which could mediate dsDNA recombination. Combined with other genetic components (e.g., counter-selection markers), derived methods could be more powerful [37]. To our knowledge, this is the first complete Red like operon identified in a Gram-positive bacterium. The operon encodes a presumptive host exonuclease inhibitor, a SSAP and a dsDNA exonuclease from 5′ to 3′. This gene arrangement is consistent with the Red operon, as well as the recently characterized Pluγβα operon from the Gram-negative bacterium *Photorhabdus luminescens* [15].

We failed to find a similar operon in *L. plantarum* JDM1, which suggested phage diversity within the species. Efficiency of Lp_0640-41-42-mediated dsDNA recombination in host strain *L. plantarum* WCFS1 was also tested, and the selection efficiency in *L. plantarum* WCFS1 was about 50 times higher (~2000 Cm^R^ colonies per ml recovery culture) than that in JDM1, partially due to the lower electroporation efficiency of JDM1 compared with that of WCFS1. These results suggested WCSF1 a better host for Lp_0640-41-42-mediated homologous recombination engineering.

In a previous study, RecTE_Psy_ was found to mediate insertion less efficiently than deletion in *Pseudomonas syringae*. Based on this finding, Zhongmeng Bao and coworkers put forward a hypothesis [38]; in this model, recombination is more favored when the length of the sequence cargo on the recombination substrate is shorter than the region of the genome being deleted, because chromosome bending is preferred to substrate bending. This idea is consistent with the observations in our *gusA* insertion assay. We obtained only 13 colonies using 1 μg phosphorothioated dsDNA substrate [an efficiency at least twofold lower than that in *glg* operon deletion, in which case the genomic region being deleted (6 kb) was even longer than the region being inserted (3 kb) in the *gusA* insertion]. However, we cannot rule out other possibilities such as inefficiency of transformation of relatively long DNA substrates (4.7 vs. 3 kb).

In *E. coli*, RecA-dependent recombination requires homologies >200 bp to achieve maximum efficiency, whereas 50 bp is enough for Red/RecET recombineering [24, 39]. Similarly in LAB, classical RecA-dependent methods use long homologies (~1 kb) for recombination [40]. In contrast to Red/RecET recombineering, Lp_0640-41-42 requires also long homologies (>1 kb) for efficient recombination. This may be due to inherent differences between the two hosts. In our preliminary experiment, Lp_0640-41-42 did not mediate recombination in *E. coli* and the Red proteins did not function in *L. plantarum* (data not shown), which indicated the recombinases from different sources have different properties.

## Conclusions

In summary, we characterized the function of the Lp_0640-41-42 protein combination originated from prophage P1 in *L. plantarum* WCFS1, which could mediate dsDNA recombination, and developed a marker-free method for genetic manipulation in *L. plantarum* accordingly. This method enabled easy screening of desired mutants and added to the genetic toolbox of LAB. This work may be instructive for identifying similar proteins for recombineering in other bacteria.

## Methods

### Bacterial strains, plasmids and media

Bacterial strains and plasmids used in this study are listed in Additional file 3: Table S1. *Lactobacillus* strains were grown in MRS broth (Oxoid) at 37 °C without shaking. *E. coli* XL1-Blue was used as the host for subcloning and other plasmid manipulations in this work and was propagated in Luria–Bertani (LB) medium at 37 °C under aeration. *Lactococcus lactis* MG1363 used for pSIP411-based vector construction was cultured statically at 30 °C in M17 broth (Oxoid) supplemented with 1 % (w/v) glucose. When needed, antibiotics were supplemented at the following concentrations: erythromycin, 5 μg/ml for lactobacilli and lactococci, and 250 μg/ml for *E. coli*; chloramphenicol, 5 μg/ml for *Lactobacillus* strains and 10 μg/ml for *E. coli*. For induction studies, sakacin P inducing peptide was obtained as a >95 % pure synthesized peptide (GenScript) and added to the growth medium at 50 ng/ml. X-Gluc (5-bromo-4-chloro-3-indolyl-β-d-glucuronic acid) was added at 100 μg/ml in the *gusA* insertion experiment to screen for prospective mutants. Gus buffer used to test the Gus enzyme activity contained: 100 mM phosphate buffer, 5 mM potassium ferricyanide, 5 mM potassium ferrocyanide, 0.5 % Triton X-100, 10 mM EDTA, 0.1 % X-Gluc, 20 % methanol, and 1 % DMSO.

### Molecular techniques

*E. coli* cells (TransGen) were transformed according to the manufacturer’s procedure. *E. coli* plasmid DNA was isolated using Plasmid Mini Kits (TIANGEN). DNA was purified using a Gel Extraction Kit (Omega) and Cycle-Pure Kit (Omega). Restriction enzymes were used according to the manufacturer’s instructions (Fermentas). PrimeSTAR polymerase (Takara) was used for PCR amplification.

Electrotransformation of lactobacilli was performed as follows. Briefly, a 2 % (v/v) inoculum from an overnight culture was inoculated into 5 ml SGMRS (MRS with 0.75 M sorbitol and 1 % glycine) and incubated at 37 °C until mid-exponential phase (OD_600_ of 0.4–0.6). Cells were centrifuged, washed twice with SM buffer (952 mM sucrose, 3.5 mM MgCl_2_), and resuspended in 80 μl of SM buffer. (The culture was induced with the inducing peptide for 40 min before centrifugation in recombination assays.) Plasmid DNA/dsDNA substrate (500 ng unless otherwise specified) was added into the suspension, and the mixture was kept on ice for 10 min before transferred to a 0.2-cm cuvette (Bio-Rad) and electroporated with a Gene Pulser (Bio-Rad, 2000 V, 25 μF, 400 Ω). After electroporation, 1 ml of SMRS broth (MRS with 0.5 M sucrose and 0.1 M MgCl_2_) was added to the cuvette and incubated at 37 °C for 3 h unless otherwise specified. Then the cells were spread on MRS agar plates containing antibiotics.

### Plasmid construction

The sequences of all oligonucleotides used are shown in Additional file 4: Table S2, and the maps of constructed plasmids are shown in Additional file 1: Figure S1. The *gnp* mutagenesis vector was constructed as follows: a 1.4-kb fragment of the upstream sequence (gnpH1) and a 1.4-kb fragment of the downstream sequence (gnpH2) of the target locus were amplified by PCR. Fragment gnpH1 was generated with primers gnp-h1f and gnp-h1r and gnpH2 with primers gnp-h2f and gnp-h2r. The plasmid skeleton with the p15A origin of replication was generated by PCR with primers p15A-f and p15A-r, using pACYC184 as the template. The chloramphenicol resistance marker was amplified from pNZ8148 using primers cat-f and cat-r containing *lox66* and *lox71* sites, respectively, on their proximal ends. Then, the four fragments, gnpH1, gnpH2, the plasmid skeleton and the *lox66*-*cat*-*lox71* cassette, sharing the overlap one by one, were assembled together under the action of T5 exonuclease (Epicentre), Phusion DNA polymerase (NEB) and *Taq* DNA ligase (NEB) in an isothermal process (Gibson assembly) [41]. The assembly mix was transformed into *E. coli* XL1-Blue followed by screening and sequencing. The *ldhD*, *glg*, *gusA* and *nagB* mutagenesis vectors were constructed in a similar way. The *gusA* gene was generated with primers gusA-f and gusA-r using pE-gusA as the template and was under control of a constitutive promoter and an artificial RBS.

Recombinase expression vectors were constructed as follows: an inducible plasmid pSIP411 (a kind gift from Lars Axelsson, Nofima, Norway) was used for gene expression in this work. pSIP411 was digested with *Nco*I and *Xho*I, followed by purification and recovery. Genes to be expressed were generated by PCR. *lp_0641* was generated with primers b-f and b-r for pLP-b construction, with b-f and b-r2 for pLP-b-exo construction, and with b-f and b-r3 for pLP-b-recE construction; *exo* was generated with exo-f and exo-r; *recE* was generated with recE-f and recE-r; *lp_0641*-*42* was generated with b-f and ba-r; *lp_0640*-*41*-*42* was generated with primers g-f and gba-r. Then, the plasmid skeleton and the respective recombinase coding genes were assembled together via Gibson assembly, and the assembly mix was transformed into *L. lactis* MG1363 followed by screening and sequencing. *lp_0640*, *lp_0641*, *lp_0642* were generated from *L. plantarum* WCFS1 genomic DNA; *exo* was generated from pTKRED; *recE* was generated from *E. coli* MG1655 genomic DNA.

### Cre-based selection marker excision

Cre was expressed on plasmid pSIP411 (pLP-cre, Ery^R^). pSIP411 was digested with *Nco*I and *Xho*I, followed by purification and recovery. The *cre* gene was amplified with primers cre-f and cre-r using pSC101-BAD-Cre-tet (a kind gift from Youming Zhang, Shandong University, China) as the template. The *cre* PCR product contained homologies with the pSIP411 vector backbone and the two fragments were assembled via Gibson assembly. As the pLP-gba plasmid is also Ery^R^, recombinants should be subjected to pLP-gba plasmid elimination before transformation of pLP-cre. Thus, mutants were cultured for 24 h in the absence of erythromycin selection. After single colony isolation, a plasmid-free strain was transformed with pLP-cre and subjected to another 24-h cultivation on induction. After single colony isolation, resultant colonies were checked by PCR for excision of the selectable marker. Finally, pLP-cre was cured by culturing the marker-free mutants for 24 h in the absence of erythromycin selection.

### Bioinformatics

To analyze the amino acid sequence of Lp_0642, the National Center for Biotechnology Information (NCBI) nonredundant (NR) database was searched using BLAST.

Potential recombinases were identified using Position-Specific Iterative BLAST (PSI-BLAST) by searching in the NCBI NR database. *L. plantarum* Lp_0640, Lp_0641 and Lp_0642 were used as initial query sequences, and five iterations were performed with standard algorithm settings.

## Additional file

10.1186/s12934-015-0344-z Plasmids constructed in this study.
10.1186/s12934-015-0344-z Inspection of *ΔldhD::gusA* mutants for the GusA activity.
10.1186/s12934-015-0344-z Strains and plasmids used in this study.
10.1186/s12934-015-0344-z Oligonucleotides used in this study.
