# Supplementary material for: Prophage recombinases-mediated genome engineering in Lactobacillus plantarum
Source: Microb Cell Fact. 2015 Oct 5;14:154. doi: 10.1186/s12934-015-0344-z (PMC4595204; doi:10.1186/s12934-015-0344-z)
Supplement: Supplementary file 1 — 10.1186/s12934-015-0344-z Plasmids constructed in this study. [file 12934_2015_344_MOESM1_ESM.docx]

**Additional file 1:**

**
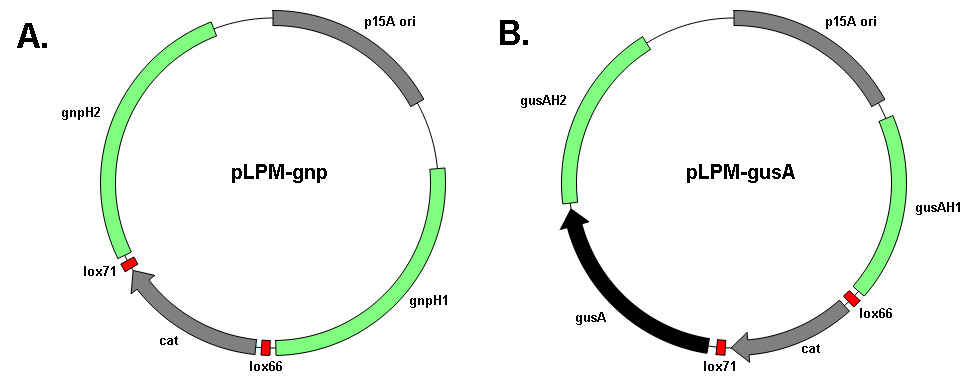
**

**Figure S1. Plasmids constructed in this study. (A)** Map of pLPM-gnp for *gnp* inactivation. This vector contained a *lox66-cat-lox71* cassette conferring chloramphenicol resistance, flanked by 1.4-kb fragments from either side of the genomic *gnp* target locus (gnpH1 and gnpH2). *lox66* and *lox71* sites were introduced for subsequent Cre-mediated removal of the *cat* marker. This vector is not replicable in *L. plantarum*. The *ldhD*, *glg*, and *nagB* mutagenesis vectors were constructed in a similar way except the different homology contents and lengths (1-kb). **(B)** Map of pLPM-gusA for *gusA* insertion. The *gusA* gene was amplified from pE-gusA using primers gusA-f and gusA-r. It was under control of a constitutive promoter and an artificial RBS, and the homology length was 1 kb.
