# Supplementary material for: Prophage recombinases-mediated genome engineering in Lactobacillus plantarum
Source: Microb Cell Fact. 2015 Oct 5;14:154. doi: 10.1186/s12934-015-0344-z (PMC4595204; doi:10.1186/s12934-015-0344-z)
Supplement: Supplementary file 2 — 10.1186/s12934-015-0344-z Inspection of ΔldhD::gusA mutants for the GusA activity. [file 12934_2015_344_MOESM2_ESM.docx]

**Additional file 2:**


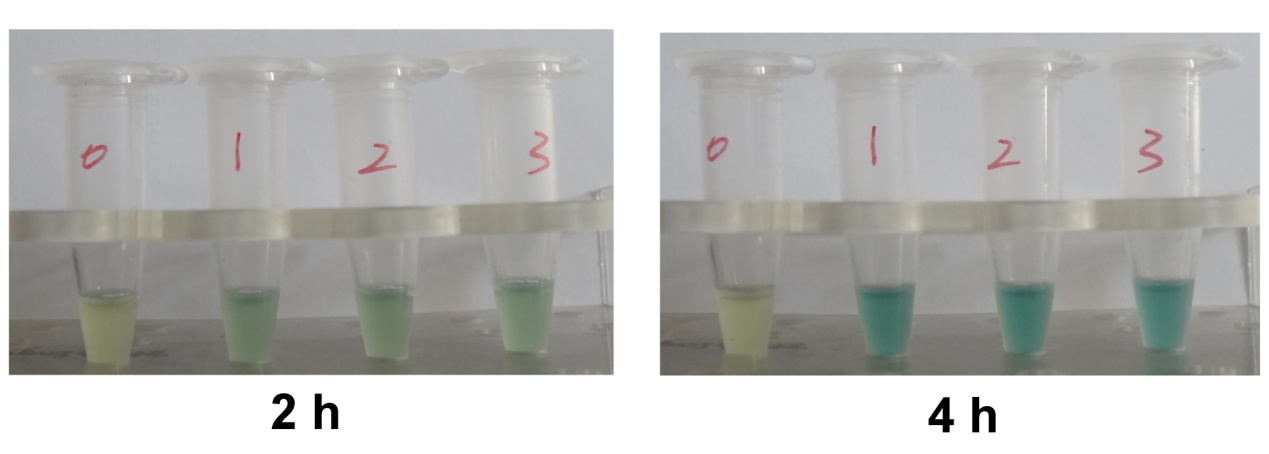


**Figure S2. Inspection of *ΔldhD::gusA* mutants for the GusA activity.** Precipitates of the wild JDM1 strain (sample 0) and three *ΔldhD::gusA* mutants (samples 1-3) were suspended in Gus buffer, and the mutants samples turned blue after 2 h while the wild strain did not. After 4 h, the phenomenon was more obvious.
