# Supplementary material for: Prophage recombinases-mediated genome engineering in Lactobacillus plantarum
Source: Microb Cell Fact. 2015 Oct 5;14:154. doi: 10.1186/s12934-015-0344-z (PMC4595204; doi:10.1186/s12934-015-0344-z)
Supplement: Supplementary file 3 — 10.1186/s12934-015-0344-z Strains and plasmids used in this study. [file 12934_2015_344_MOESM3_ESM.docx]

**Additional file 3:**

**Table S1. Strains and plasmids used in this study**

| Strain or plasmid | Relevant characteristics | Reference |
| --- | --- | --- |
| Strains |  |  |
| *Lactobacillus plantarum* JDM1 | wild type | [[1](#_ENREF_1)] |
| *Lactobacillus plantarum* WCFS1 | wild type | [[2](#_ENREF_2)] |
| *Escherichia coli* XL1-Blue | subcloning host | Transgen |
| *Lactococcus lactis* MG1363 | subcloning host | [[3](#_ENREF_3)] |
|  |  |  |
| Plasmids |  |  |
| pACYC184 | source of p15A origin of replication | lab stock |
| pNZ8148 | source of chloramphenicol-resistance gene *cat* | lab stock |
| pTKRED | pSC101ori, red recombinases, Spc^R^ | [[4](#_ENREF_4)] |
|  |  |  |
| pSIP411 | *spp*-based inducible expression vector; SH71_rep_; Ery^R^; P_orfX_:: *gusA* | [[5](#_ENREF_5)] |
| pE-gusA | 256_rep_; Ery^R^, *gusA* under control of a constitutive promoter | lab stock |
| pLP-b | pSIP411derivative, P_orfX_:: *lp_0641* | This study |
| pLP-ba | pSIP411 derivative, P_orf_X:: *lp_0641-lp_0642* | This study |
| pLP-gba | pSIP411 derivative, P_orfX_:: *lp_0640-lp_0641-lp_0642* | This study |
| pLP-b-exo | pSIP411 derivative, P_orfX_:: *lp_0641-exo* | This study |
| pLP-b-recE | pSIP411 derivative, P_orfX_:: *lp_0641-recE* | This study |
| pLP-cre | pSIP411 derivative, P_orfX_:: *cre* | This study |
| pLPM-gnp | mutagenesis vector for *gnp* deletion; Cm^R^; with 1.4 kb flanks | This study |
| pLPM-ldhD | mutagenesis vector for *ldhD* deletion; Cm^R^; with 1 kb flanks | This study |
| pLPM-gusA | mutagenesis vector for *gusA* insertion; Cm^R^; with 1 kb flanks | This study |
| pLPM-glg | mutagenesis vector for *glg* deletion; Cm^R^; with 1 kb flanks | This study |
| pLPM-nagB | mutagenesis vector for *nagB* deletion; Cm^R^; with 1 kb flanks | This study |

**References**

1. Zhang ZY, Liu C, Zhu YZ, Zhong Y, Zhu YQ, Zheng HJ, Zhao GP, Wang SY, Guo XK: **Complete genome sequence of *Lactobacillus plantarum* JDM1.** *J Bacteriol* 2009, **191:**5020-5021.

2. Kleerebezem M, Boekhorst J, van Kranenburg R, Molenaar D, Kuipers OP, Leer R, Tarchini R, Peters SA, Sandbrink HM, Fiers MW, et al: **Complete genome sequence of *Lactobacillus plantarum* WCFS1.** *Proc Natl Acad Sci U S A* 2003, **100:**1990-1995.

3. Wegmann U, O'Connell-Motherway M, Zomer A, Buist G, Shearman C, Canchaya C, Ventura M, Goesmann A, Gasson MJ, Kuipers OP, et al: **Complete genome sequence of the prototype lactic acid bacterium *Lactococcus lactis* subsp. *cremoris* MG1363.** *J Bacteriol* 2007, **189:**3256-3270.

4. Kuhlman TE, Cox EC: **Site-specific chromosomal integration of large synthetic constructs.** *Nucleic Acids Res* 2010, **38:**e92.

5. Sorvig E, Mathiesen G, Naterstad K, Eijsink VG, Axelsson L: **High-level, inducible gene expression in *Lactobacillus sakei* and *Lactobacillus plantarum* using versatile expression vectors.** *Microbiology* 2005, **151:**2439-2449.
