# Supplementary material for: Prophage recombinases-mediated genome engineering in Lactobacillus plantarum
Source: Microb Cell Fact. 2015 Oct 5;14:154. doi: 10.1186/s12934-015-0344-z (PMC4595204; doi:10.1186/s12934-015-0344-z)
Supplement: Supplementary file 4 — 10.1186/s12934-015-0344-z Oligonucleotides used in this study. [file 12934_2015_344_MOESM4_ESM.docx]

**Additional file 4:**

**Table S2. Oligonucleotides used in this study**

| Primer | Sequence(5'-3')^a^ |
| --- | --- |
| p15A-f | gcgctagcggagtgtatactggc |
| p15A-r | ACAACTTATATCGTATGGGGCTG |
| cat-f | ccTaccgttcgtataatgtatgctatacgaagttatACGAAAGTCGACGGCAATAGTTAC |
| cat-r | GGTACCGTTCGTATAGCATACATTATACGAAGTTATCTGTAATATAAAAACCTTCTTCAAC |
| gnp-h1f | gtcagccccatacgatataagttgtCTTGACCGATGGGAGATGTGAATGA |
| gnp-h1r | GATAACTTCGTATAGCATACATTATACGAACGGTAGGAATTAACTGACTCCCTCTTTG |
| gnp-h2f | GAtaacttcgtataatgtatgctatacgaacggtaccTCGGCGAAAACAAGGCTGATGCGGT |
| gnp-h2r | GCCAGTATACACTCCGCTAGCGCTTACCCCATCCTATGCAGCAATCGC |
| gnp-testA | TTAACTTGATAAGCCCCGTTCGGTG |
| gnp-testB | ACATTGAGCCAGACCGTCGCTACTT |
| ldhD-h1f | gtcagccccatacgatataagttgtTTTGGCGATTGCACGGCATGAACTG |
| ldhD-h1r | GATAACTTCGTATAGCATACATTATACGAACGGTAGGACATCGGCACCGTCGAAGCCTTTAG |
| ldhD-h2f | GAtaacttcgtataatgtatgctatacgaacggtaccCACAAGTTAAGTTTGACTAATCTCG |
| ldhD-h2r | GCCAGTATACACTCCGCTAGCGCCGTTAGCACCCAGCAAAGTCCACAT |
| ldhD-testA | TGCGTTCTCACTGGATGCTGTTCT |
| ldhD-testB | AAATCACCGTTCACTGCTGCCTTA |
| glg-h1f | ggaccatggctaattcccatTACAGTCCAGCAAAGGCTAAACAG |
| glg-h1r | TAGCATACATTATACGAACGGTAGGTTAGTGCGCTTGGTTGCCAGTCAT |
| glg-h2f | taatgtatgctatacgaacggtaccCTTGGCAGATTATGGCATTGGCTAC |
| glg-h2r | GCCAGTATACACTCCGCTAGCGCCAACGGATCAAACGTCAGTAAGAA |
| glg-testA | TGCTGGCAATACCAAGAACCCGACAA |
| glg-testB | AGCGCACGCGGATTATTACGAACA |
| gusA-f | Gtataatgtatgctatacgaacggtaccaagcttttataaaaagatgttgacag |
| gusA-r | GCAGTATAGTCCTTTTGTTGGTATGAATTCTCATTGTTTGCCTCCCTGC |
| gusA-h2f | ATACCAACAAAAGGACTATACTGC |
| gusA-h2r | GCCAGTATACACTCCGCTAGCGCTCGAGATTAGTCAAACTTAACTTG |
| gus-testB | CCACAGCCGTGCAACCAGCTCTAT |
| nagB-h1f | gtcagccccatacgatataagttgtACCTCGTGTCCGTTGAATTTCTGG |
| nagB-h1r | GTATAGCATACATTATACGAACGGTAGGCGGCAGCTTGATCCTTTACTACGA |
| nagB-h2f | gtataatgtatgctatacgaacggtaccCGTTACCGTTATCATTGACGAAGC |
| nagB-h2r | GCCAGTATACACTCCGCTAGCGCATGGATCTCAACGATACCGACAAT |
| cre-f | AATAATATATAGGAGTATGATTCCCATGTCCAATTTACTGACCGTAC |
| cre-r | CGAACCCGGGGTACCGAATTCCTCGAGCTAATCGCCATCTTCCAGCAGG |
| b-f | TAAAATAATATATAGGAGTATGATTCCCATGAGTAATGAGCTAGTTACGATG |
| b-r | GGGTACCGAATTCCTCGAGTCTAGATTAGCTGGCGTCAAAGTCTCCGAGG |
| b-r2 | ATATATAAACTCCTTACTTGCGAATCTGTTATTAGCTGGCGTCAAAGTCTCCGAGG |
| b-r3 | ATATATAAACTCCTTACTTGCGAATCTGTTATTAGCTGGCGTCAAAGTCTCCGAGG |
| ba-r | GGGTACCGAATTCCTCGAGTCTAGATCAATCTATGAGTAAGTCGTCTGCAC |
| g-f | TAAAATAATATATAGGAGTATGATTCCCATGATACCAGCACAGGCAGGTTTAAAC |
| exo-f | TAACAGATTCGCAAGTAAGGAGTTTATATATatgacaccggacattatcctgcagcg |
| exo-r | GGGTACCGAATTCCTCGAGTCTAGATCATCGCCATTGCTCCCCAAATACAAAA |
| recE-f | TAACAGATTCGCAAGTAAGGAGTTTATATATatgagcacaaaaccactcttcctg |
| recE-r | GGGTACCGAATTCCTCGAGTCTAGATTAGTCATTTGCATATTCCTTAGCCC |

1. Underlined sequences indicate homology arms for Gibson assembly.
